# High on-aspirin treatment platelet reactivity and restenosis after percutaneous coronary intervention: results of the Intracoronary Stenting and Antithrombotic Regimen-ASpirin and Platelet Inhibition (ISAR-ASPI) Registry

**DOI:** 10.1007/s00392-023-02161-z

**Published:** 2023-02-14

**Authors:** Katharina Mayer, Gjin Ndrepepa, Mira Schroeter, Christopher Emmer, Isabell Bernlochner, Stefanie Schüpke, Senta Gewalt, Raphaela Hilz, John Joseph Coughlan, Alp Aytekin, Clarissa Heyken, Tanja Morath, Heribert Schunkert, Karl-Ludwig Laugwitz, Dirk Sibbing, Adnan Kastrati

**Affiliations:** 1 Deutsches Herzzentrum München, Cardiology and Technische Universität München, Lazarettstr. 36, 80636 Munich, Germany; 2 Medizinische Klinik and Poliklinik Innere Medizin I (Kardiologie, Angiologie, Pneumologie), Klinikum rechts der Isar, Technische Universität München, Munich, Germany; 3 Klinik der Universität München, Cardiology, Ludwig-Maximilians-Universität, Munich, Germany; 4 Privatklinik Lauterbacher Mühle am Ostersee, Iffeldorf und Ludwig-Maximilians-Univerität, Munich, Germany; 5 DZHK (German Center for Cardiovascular Research), Partner Site Munich Heart Alliance, Munich, Germany

**Keywords:** High platelet reactivity, Aspirin, Restenosis, Target lesion revascularization, Percutaneous coronary intervention

## Abstract

**Objective:**

The aim of this study was to assess the association between high on-aspirin treatment platelet reactivity (HAPR) and the subsequent risk of restenosis after percutaneous coronary intervention (PCI) with predominantly drug-eluting stents.

**Background:**

The association between HAPR and subsequent risk of restenosis after PCI is unclear.

**Methods:**

This study included 4839 patients undergoing PCI (02/2007–12/2011) in the setting of the Intracoronary Stenting and Antithrombotic Regimen-ASpirin and Platelet Inhibition (ISAR-ASPI) registry. Platelet function was assessed with impedance aggregometry using the multi-plate analyzer immediately before PCI and after intravenous administration of aspirin (500 mg). The primary outcome was clinical restenosis, defined as target lesion revascularization at 1 year. Secondary outcomes included binary angiographic restenosis and late lumen loss at 6- to 8-month angiography.

**Results:**

The upper quintile cut-off of platelet reactivity measurements (191 AU × min) was used to categorize patients into a group with HAPR (platelet reactivity > 191 AU × min; *n = *952) and a group without HAPR (platelet reactivity ≤ 191 AU × min; *n = *3887). The primary outcome occurred in 94 patients in the HAPR group and 405 patients without HAPR (cumulative incidence, 9.9% and 10.4%; HR = 0.96, 95% CI 0.77–1.19; *P = *0.70). Follow-up angiography was performed in 73.2% of patients. There was no difference in binary restenosis (15.2% vs. 14.9%; *P = *0.79) or late lumen loss (0.32 ± 0.57 vs. 0.32 ± 0.59 mm; *P = *0.93) between patients with HAPR versus those without HAPR.

**Conclusions:**

This study did not find an association between HAPR, measured at the time of PCI, and clinical restenosis at 1 year after PCI.

**Graphical abstract:**

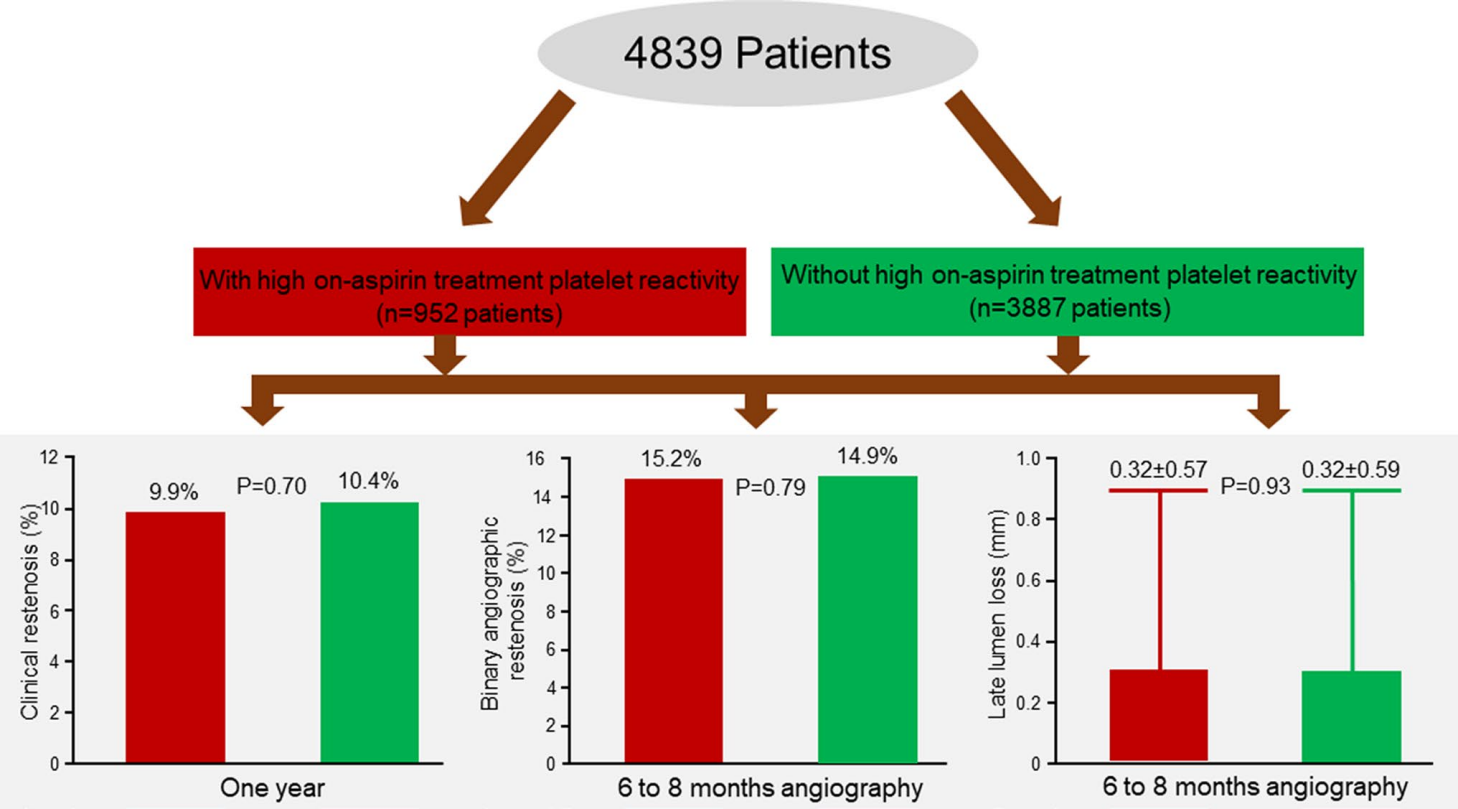

## Introduction

In patients treated with percutaneous coronary intervention (PCI), drug-eluting stents (DES) have reduced the rates of restenosis and target lesion revascularization (TLR) compared with bare-metal stents (BMS) [1–3]. However, in-stent restenosis (ISR) after DES implantation can occur and it presents a clinical challenge in terms of therapy [4–6]. Given the principal role of platelets in acute thrombosis, pharmacological platelet inhibition is mandatory in PCI [7]. Early platelet response and platelet involvement in the cellular processes following PCI play an important role in the development of neo-intimal proliferation in response to vascular injury following PCI. Neo-intimal proliferation may subsequently lead to restenosis [8–10]. Dual antiplatelet therapy (DAPT) with aspirin and a P2Y_12_ inhibitor is a guideline-recommended therapy for patients undergoing PCI [8–10]. The duration of DAPT varies depending on several factors, including the clinical presentation [10, 11]. Patients treated with PCI for ISR may benefit from prolonged DAPT [12]. High on-clopidogrel treatment platelet reactivity (HCPR) has been linked to a higher risk for ischemic events after PCI [13], and the evidence remains limited with respect to an association between HCPR subsequent ISR [14, 15]. Likewise, there are conflicting results with respect to an association between high on-aspirin treatment platelet reactivity (HAPR) and restenosis after PCI [16–18]. Reasoning from these facts, we undertook this study to investigate whether there is an association between HAPR and the subsequent risk of restenosis in patients undergoing PCI with predominantly DES implantation.

## Methods

### Patients

The study included 4839 patients undergoing PCI in 2 university hospitals (Deutsches Herzzentrum München and Medizinische Klinik and Poliklinik Innere Medizin I, Klinikum rechts der Isar, Technische Universität München, both in Munich, Germany) between February 2007 and December 2011. The source sample included 7090 patients enrolled in the Intracoronary Stenting and Antithrombotic Regimen-ASpirin and Platelet Inhibition (ISAR-ASPI) registry between February 2007 and May 2013 [19]. To be included in the current analysis, patients had to have been pretreated with aspirin, had platelet aggregation and clinical outcome (bleeding and ischemic events) data and Quantitative Coronary Angiography (QCA) data available. All patients received aspirin (an intravenous dose of 500 mg) and pre-treatment with an ADP receptor antagonist before the PCI procedure. In the post-PCI period, DAPT and other medications were prescribed as per standard practice. Aspirin, 100 mg twice daily, was recommended for an indeterminate duration. All patients had platelet function test measurements for HAPR performed immediately before the PCI procedure. Patients presenting with chronic coronary syndromes, unstable angina, ST-elevation myocardial infarction (STEMI), or non-ST-elevation myocardial infarction (NSTEMI) were included. Patients with cardiogenic shock and those who developed stent thrombosis at the index PCI were excluded. Data on mortality and stent thrombosis in the ISAR-ASPI registry have already been published [19]. The study conforms to the Declaration of Helsinki.

Cardiovascular risk factors were defined according to the generally accepted criteria. Global left ventricular ejection fraction was measured using the area–length method on left ventricular angiograms. Patient’s weight and height were measured during the index hospitalization and used to calculate the body mass index. Glomerular filtration rate was estimated using the Modification of Diet in Renal Disease (MDRD) Study equation.

### Blood sampling and platelet function testing

Blood for platelet function testing was sampled into 4.5 ml lepirudin-containing blood vials (25 µg/ml, Refludan, Dynabyte, Munich, Germany). Blood for platelet function testing was collected from the arterial sheath before the start of PCI and after intravenous administration of aspirin. Arachidonic acid (AA) and adenosine diphosphate (ADP)-induced platelet aggregation were measured with MEA on an impedance aggregometer (Multiplate analyzer), and results obtained were expressed as arbitrary aggregation units plotted against time (AU × min). Impedance aggregometry using the Multiplate analyzer (Roche Diagnostics, Basel, Switzerland) was used for quantitative analysis of platelet function (inhibition) triggered by arachidonic acid (AA). The increase of impedance caused by platelet attachment to the incorporated electrodes is converted into aggregation units (AU) and plotted against time (AU × min). The materials used for impedance aggregometry including the activator substance AA originated from the manufacturer Roche Diagnostics (Basel, Switzerland).

### Study outcome measures and definitions

HAPR was defined as having a platelet aggregation value in the upper quintile (20%). The primary endpoint was clinical restenosis, defined as a target lesion revascularization (TLR) performed at follow-up angiography for a lesion with a diameter stenosis > 70% (irrespective of the clinical symptoms) or for a lesion with a diameter stenosis ≥ 50% with clinical symptoms or signs indicative of myocardial ischemia [20]. The primary endpoint was assessed at 1 year. Secondary endpoints consisted of binary angiographic restenosis (BAR) and late lumen loss (LLL) at 6–8-month follow-up angiography. The BAR was defined as a stenosis ≥ 50% lumen obstruction on control angiography. The LLL was calculated as the difference between the post-stenting minimal lumen diameter (MLD) and the MLD measured at follow-up coronary angiography. Angiographic assessment was performed offline by blinded personnel of the Quantitative Coronary Angiography (QCA) core laboratory using a validated automated edge detection system (CMS version 7.1, Medis Medical Imaging Systems, Leiden, The Netherlands). In-hospital bleeding, defined according to the Thrombolysis in Myocardial Infarction (TIMI) group criteria was also assessed.

### Follow-up

Patients were hospitalized for at least 2 days after the index PCI. The post-discharge follow-up included telephone interviews at 30 days, 6 months and 1 year. Patients who had clinical symptoms underwent a comprehensive clinical, electrocardiographic, and laboratory assessment in the outpatient clinic. As a standard practice in our institutions, at the time of patient’s inclusion in the registry, all patients were scheduled to undergo coronary angiography 6 months after the procedure or whenever they showed symptoms or signs of myocardial ischemia. The data were prospectively collected and saved in an electronic database by personnel of the ISAResearch Center. Information from referring physicians and hospital readmissions was also incorporated into the database.

### Statistical methods

Continuous data are presented using mean ± standard deviation (SD) or median [25th–75th] percentiles. The distribution pattern of continuous data was assessed using the Kolmogorov–Smirnov test. Categorical data are presented as counts and proportions (%). Continuous variables were compared using the *t* test or Wilcoxon rank-sum test, as appropriate. Categorical data were compared using the chi-square test. Cumulative incidence of the primary outcome was calculated using the Kaplan–Meier method, and the differences in event-free survival were compared with the log-rank test. The Cox proportional hazards model was performed to assess the correlates of the primary outcome. The multiple linear regression model was used to assess the association of HAPR with BAR. The following variables were entered into the models: HAPR, age, sex, body mass index, arterial hypertension, hypercholesterolemia, smoking, clinical presentation, glomerular filtration rate, baseline C-reactive protein, ADP-induced platelet aggregation values, stent type, plus HAPR-by-clinical presentation interaction term. All analyses were performed using the R Statistical Software (R Statistical Software, Foundation for Statistical Computing, Vienna, Austria). A two tailed *P* value of < 0.05 was considered to confer statistical significance.

## Results

### Patients

The upper quintile cut-off of platelet reactivity measurements in our study was 191 AU × min. Based on the upper quintile cut-off of platelet reactivity measurements, patients were categorized into 2 groups: a group with HAPR (platelet reactivity ≥ 191 AU × min; *n = *952 patients) and a group without HAPR (platelet reactivity < 191 AU × min; *n = *3887 patients). Baseline data are shown in Table 1. Patients with versus without HAPR appear to differ with respect to several characteristics. Patients with HAPR were less likely to have arterial hypertension and hypercholesterolemia and had lower body mass index, lower glomerular filtration rate and higher levels of C-reactive protein compared with patients without HAPR. Overall, 2863 patients (59.2%) presented with chronic coronary syndromes and 1976 patients (40.8%) presented with acute coronary syndromes. The angiographic and the procedural characteristics (lesion-based analysis) are shown in Table 2. The majority of lesions in both groups (> 95%) were treated with a DES at the index PCI.

**Table 1:** Baseline characteristics

Characteristic	AA-induced platelet aggregation		*P* value
	With HAPR (*n = *952)	Without HAPR (*n = *3887)	
Age (years)	68.5 ± 11.1	67.8 ± 10.8	0.07
Women	201 (21.1)	929 (23.9)	0.07
Diabetes mellitus	268 (28.2)	1124 (28.9)	0.64
On insulin therapy	89 (9.3)	347 (8.9)	0.68
Arterial hypertension	372 (39.1)	1821 (46.8)	< 0.001
Hypercholesterolemia	636 (66.8)	2855 (73.4)	< 0.001
Active smoker	139 (14.6)	665 (17.1)	0.06
Body mass index (kg/m^2^)	27.1 ± 4.2	27.7 ± 4.4	0.002
Left ventricular ejection fraction	52.6 ± 12.2	53.6 ± 11.2	0.11
Serum creatinine (mg/dl)	1.1 ± 0.4	1.1 ± 0.7	0.10
GFR (ml /min)	84.2 ± 34.3	88.5 ± 35.4	< 0.001
C-reactive protein (mg/L)	13.7 ± 35.4	8.1 ± 22.8	0.001
ADP-induced platelet aggregation (AU x min)	367.5 ± 272.7	259.6 ± 223.1	< 0.001
Previous myocardial infarction	256 (26.9)	1020 (26.2)	0.68
Previous bypass surgery	138 (14.5)	566 (14.6)	0.96
Coronary artery disease			0.67
1-vessel disease	163 (17.1)	619 (15.9)	
2-vessel disease	253 (26.6)	1043 (26.8)	
3-vessel disease	536 (56.3)	2225 (57.2)	
Number of lesions	1.8 ± 1.0	1.8 ± 0.9	0.98
Clinical presentation			0.05
ST-segment elevation MI	104 (10.9)	367 (9.4)	
Non-ST-segment elevation MI	50 (5.3)	149 (3.8)	
Unstable angina	234 (24.6)	1072 (27.6)	
Stable angina	564 (59.2)	2299 (59.2)	

Data are mean ± standard deviation or number of patients (%)

*AA* arachidonic acid; *ADP *adenosine diphosphate; *AU* aggregation unit; *GFR* glomerular filtration rate; *HAPR* high on-aspirin treatment platelet reactivity; *MI* myocardial infarction

**Table 2:** Angiographic and procedural characteristics (lesion-based analysis)

Characteristic	AA-induced platelet aggregation		*P* value
	With HAPR (1695 lesions)	Without HAPR (6814 lesions)	
Left main coronary artery	86 (5.1)	353 (5.2)	
Left anterior descending coronary artery	692 (40.8)	2702 (39.7)	
Left circumflex coronary artery	422 (24.9)	1711 (25.1)	
Right coronary artery	466 (27.5)	1898 (27.9)	
Venous bypass graft	29 (1.7)	150 (2.2)	
Complex (type B2 or C) lesions	1242 (73.3)	5058 (74.2)	0.42
Chronic occlusions	96 (5.7)	371 (5.4)	0.72
Ostial lesions	392 (23.1)	1610 (23.6)	0.66
Bifurcation lesions	494 (29.1)	2022 (29.7)	0.67
Type of intervention			0.02
Placement of drug-eluting stent	1626 (95.9)	6497 (95.4)	
Placement of bare-metal stent	23 (1.4)	58 (0.9)	
Balloon angioplasty	46 (2.7)	259 (3.8)	
Lesion length (mm)	16.0 ± 9.7	16.2 ± 25.5	0.88
Reference diameter (mm)	2.8 ± 0.6	2.9 ± 0.6	0.04

Data are number of lesions (%) or mean ± standard deviation

*AA* arachidonic acid; *HAPR* high on-aspirin treatment platelet reactivity

### Primary endpoint (clinical restenosis)

The primary endpoint occurred in 94 of 952 patients with HAPR and 405 of 3887 patients without HAPR (cumulative incidence, 9.9% and 10.4%, respectively, hazard ratio [HR] = 0.96, 95% confidence interval [CI] 0.77–1.19; *P = *0.70). In-hospital bleeding events (TIMI major or minor) occurred in 68 of 952 patients with HAPR and 232 of 3887 patients without HAPR (7.1% vs. 6.0%; *P = *0.18).

The Cox proportional hazards model (see methods for variables we adjusted for) showed that HAPR was not an independent correlate of the primary endpoint (adjusted hazard ratio = 0.98, 95% confidence interval 0.78–1.23; *P = *0.85, calculated per HAPR quintile). There was no HAPR-by-clinical presentation (chronic coronary syndromes or acute coronary syndromes) interaction with respect to the primary endpoint (*P* for interactio*n = *0.32).

### Secondary endpoints (angiographic outcomes)

**Fig. 1:**
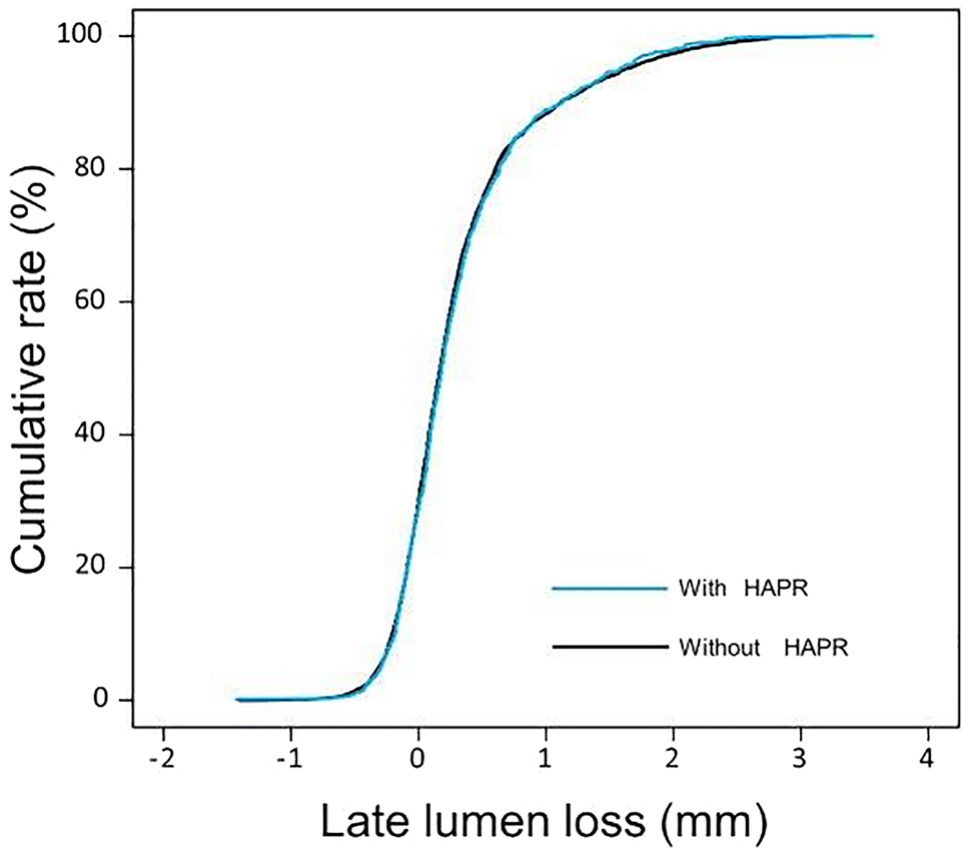
Cumulative distribution curves of late lumen loss in patients with (blue line) and without high on-aspirin treatment platelet reactivity (HAPR) (black line)

Repeat coronary angiography was performed in 73.2% of patients at 6–8 months after the index PCI. The angiographic outcomes for both groups are detailed in Table 3. There were no significant differences in the rates of BAR (15.2% vs. 14.9%; *P = *0.79) or the degree of LLL (0.32 ± 0.57 vs. 0.32 ± 0.59 mm; *P = *0.93; Fig. 1) between the two groups. HAPR was also not independently associated with the increased risk of BAR (adjusted odds ratio = 0.99 [0.97–1.01], *P = *0.30, calculated per HAPR unit). There was no HAPR-by-clinical presentation (chronic coronary syndromes or acute coronary syndromes) interaction with respect to the BAR (*P* for interactio*n = *0.14).

**Table 3:** Angiographic outcomes at 6- to 8-month repeat coronary angiography

Characteristic	AA-induced platelet aggregation		*P* value
	With HAPR (1085 lesions)	Without HAPR (5216 lesions)	
Binary restenosis (%)	165 (15.2)	777 (14.9)	0.79
Late lumen loss (mm)	0.32 ± 0.57	0.32 ± 0.59	0.93
Late lumen loss (mm)^a^	0.18 [− 0.04 to 0.50]	0.17 [− 0.04 to 0.49]	0.46
Diameter stenosis (%)	32.8 ± 18.4	32.8 ± 18.6	0.92
Minimal lumen diameter (mm)	1.99 ± 0.69	2.01 ± 0.71	0.54

Data are number of lesions (%) or mean ± standard deviation

*AA* arachidonic acid; *HAPR* high on-aspirin treatment platelet reactivity

^a^Late lumen loss data in this line are median with 25th–75th percentiles

## Discussion

The main findings of this study may be summarized as follows: (1) HAPR assessed at the time of PCI was not associated with increased risk of clinical restenosis (defined as TLR) at 1 year after DES implantation. (2) There was no association between HAPR and angiographic results including BAR and LLL on repeat coronary angiography at 6–8 months after PCI.

Restenosis may be secondary to neointimal hyperplasia as a consequence of vascular injury during PCI. PCI induces vascular injury and exposes sub-endothelial tissue inducing an adhesive platelet response. Platelet adhesion at the site of PCI-induced vascular injury can lead to local thrombus formation, which may be an important step in the development of restenosis. Platelet recruitment in the area of the neo-intimal injury can promote proliferation and migration of smooth muscle cells [21–23]. Preclinical studies have suggested a relationship between an early platelet response and subsequent development of restenosis. However, whether antithrombotic therapy can mitigate this response and reduce the risk of restenosis remains unclear [24–27]. Several pharmaco-therapeutic strategies were tested in this regard, including warfarin [28], heparin [29], bivalirudin [30], ticlopidine [31], thromboxane A_2_ blockers [16, 32], and a combination of aspirin and dipyridamole [33]. Some of these studies have demonstrated a reduction in early thrombotic events post PCI but not a reduction in restenosis rates [16, 30, 33].

HCPR is associated with a higher risk for thrombotic events after PCI [13]. However, clinical studies assessing the antirestenotic efficacy of clopidogrel have reported conflicting results [14, 15]. Schulz et al. [15] showed that HCPR was not associated with an increased risk of restenosis in 1608 patients with HCPR after DES implantation. Conversely, Fu et al. [14] showed that HCPR was independently associated with the increased risk of ISR. A sub-study of the Prolonging Dual Antiplatelet Treatment After Grading Stent-Induced Intimal Hyperplasia (PRODIGY) trial suggested that patients treated with repeat PCI for ISR may benefit from long-term (24 months) administration of DAPT [12].

Aspirin is a well-established therapy for patients with cardiovascular disease. The beneficial effects of aspirin in terms of reduction of vascular death, myocardial infarction, or stroke in patients with stable coronary heart disease or acute coronary syndromes are recognized and current guidelines recommend aspirin as standard maintenance therapy in these patients [8]. However, the impact of HAPR on clinical outcomes is less clear than the impact of HCPR. A previous publication from the ISAR-ASPI registry showed an association between HAPR and increased risk of death or stent thrombosis at 1 year after DES implantation [19]. Conversely, a prospective (Assessment of Dual AntiPlatelet Therapy with Drug-Eluting Stents) registry of 8665 patients did not show a significant association between HAPR and the risk for stent thrombosis, myocardial infarction or death. However, the study showed an inverse association between HAPR and bleeding with a significant 35% lower adjusted risk for bleeding in patients with HAPR after DES implantation [34].

Few small studies have investigated the association between HAPR and the risk for ISR [16–18]. A randomized study of 216 patients undergoing angioplasty for a previously untreated native coronary artery lesions showed that aspirin reduced the rate of restenosis at 6 months in lesion-based analysis (25% vs. 38%) compared with placebo demonstrating a small benefit of aspirin in reducing the restenosis after angioplasty [17]. LLL was also less in aspirin-treated patients compared with placebo-treated patients (16 ± 22% vs. 22 ± 25%) [17]. Savage et al. [16] reported that aspirin protected against late ischemic events after angioplasty even though angiographic restenosis was not significantly reduced. Finally, Pamuckcu et al. [18] assessed the association between aspirin resistance and ISR in 204 patients with coronary artery disease after coronary stent implantation. Aspirin resistance was higher (31.3% vs. 10.7%) in patients who developed ISR compared with those who did not develop ISR. The current investigation may be the first large-scale study assessing the association between HAPR and clinical and angiographic parameters of restenosis after DES implantation. The data showed that there was no difference in the occurrence of the primary endpoint, 1-year rates of clinical restenosis between patients with and without HAPR. In addition, the rate of BAR and the degree of LLL on coronary angiography at 6- to 8-month post-PCI did not differ among patients with or without HAPR.

## Limitations

The current study has several limitations. This is an observational retrospective analysis of registry-based data. As such, it has the limitations inherent to this type of studies. In addition, baseline (off-treatment) platelet function values are missing and therefore we could not perform an assessment of aspirin response. However, at the time of the study, only the Multiplate analyzer was used as part of routine platelet function testing following PCI. Thus, we acknowledge the lack of other assays to assess platelet reactivity such as calibrated automated thrombogram (CAT) assay. Therefore, it is unclear whether our findings can be extrapolated to other platelet function testing devices. At the time of patients’ inclusion in the registry, a maintenance dose of aspirin of 100 mg twice daily was recommended as per local practice. Although it does not reflect current recommendations with respect to the maintenance dose of aspirin after PCI, we do not believe that the higher dose of aspirin as used in current study has had an impact on the main study outcomes. Follow-up angiography was not performed in approximately 27% of the patients. Finally, the study findings were based on a single platelet reactivity measurement, and consequently we have no follow-up or serial data on the chronic platelet response.

## Conclusions

In patients with coronary artery disease, HAPR assessed at the time of PCI was not associated with increased risk of clinical restenosis (defined as TLR) at 1-year post-PCI compared to patients without HAPR. In addition, the risk of binary angiographic restenosis and the degree of late lumen loss on 6- to 8-month repeat coronary angiography were similar between the two groups. Further prospective studies may be useful to further elucidate any association between the degree of peri-procedural platelet inhibition and the subsequent risk of restenosis after DES implantation.

## Data Availability

The authors confirm that the data used for this study might be shared after reasonable request and submission of an analysis plan to the corresponding author.

## Abbreviations

AA: Arachidonic acid
ADP: Adenosine diphosphate
AU: Aggregation unit
BAR: Binary angiographic restenosis
BMS: Bare-metal stent
CI: Confidence interval
DES: Drug-eluting stent
HAPR: High on-aspirin treatment platelet reactivity
HCPR: High on-clopidogrel treatment platelet reactivity
HR: Hazard ratio
IRS: In-stent restenosis
LLL: Late lumen loss
MI: Myocardial infarction
MLD: Minimal lumen diameter
NSTEMI: Non-ST-elevation myocardial infarction
PCI: Percutaneous coronary intervention
ST: Stent thrombosis
STEMI: ST-elevation myocardial infarction
TIMI: Thrombolysis in Myocardial Infarction
TLR: Target lesion revascularization
QCA: Quantitative Coronary Angiography
